# Mechanisms of *Tripterygium wilfordii* Hook F on treating rheumatoid arthritis explored by network pharmacology analysis and molecular docking

**DOI:** 10.1515/med-2024-0967

**Published:** 2024-05-30

**Authors:** Ni Mao, Xi Xie

**Affiliations:** Department of Rheumatology, The Second Xiangya Hospital of Central South University, Changsha, Hunan, 410011, China; Clinical Medical Research Center for Systemic Autoimmune Diseases in Hunan Province, Changsha, Hunan, China

**Keywords:** network pharmacology, rheumatoid arthritis, *Tripterygium wilfordii* Hook F, inflammatory cytokine, chemotaxis, angiogenesis, molecular docking

## Abstract

**Background:**

Rheumatoid arthritis (RA) is a chronic inflammatory and disabling disease that imposes significant economic and social costs. *Tripterygium wilfordii* Hook F (TwHF) has a long history of use in traditional Chinese medicine for treating joint disorders, and it has been shown to be cost-effective in treating RA, but its exact mechanism is unknown.

**Objective:**

The goal of the network pharmacology analysis and molecular docking was to investigate the potential active compounds and associated anti-RA mechanisms of TwHF.

**Methods:**

TCMSP and UniProt databases were searched for active compounds and related targets of TwHF. PharmGKB, DrugBank, OMIM, TTD, and the Human Gene Databases were used to identify RA-related targets. The intersected RA and TwHF targets were entered into the STRING database to create a protein–protein interaction network. R software was used for gene ontology (GO) and Kyoto Encyclopedia of Genes and Genomes (KEGG) enrichment analyses. Molecular docking technology was used to analyze the optimal effective components from TwHF for docking with the selected target gene.

**Results:**

Following screening and duplicate removal, a total of 51 active compounds and 96 potential targets were chosen. The PPI network revealed that the target proteins are CXCL8, CXCL6, STAT3, STAT1, JUN, PPARG, TP53, IL14, MMP9, VEGFA, RELA, CASP3, PTGS2, IFNG, AKT1, FOS, ICAM1, and MAPK14. The results of the GO enrichment analysis focused primarily on the response to lipopolysaccharide, the response to molecules of bacterial origin, and the response to drugs. The KEGG results indicated that the mechanisms were closely related to lipid and atherosclerosis, chemical carcinogenesis-receptor activation, Kaposi sarcoma-associated, herpesvirus infection, hepatitis B, fluid shear stress and atherosclerosis, IL-17 signaling pathways, Th17-cell differentiation, and so on, all of which are involved in angiogenesis, immune cell chemotaxis, and inflammatory responses. Molecular docking results suggested that triptolide was the appropriate PTGS1, PTGS2, and TNF inhibitors.

**Conclusion:**

Our findings provide an essential role and basis for further immune inflammatory studies into the molecular mechanisms of TwHF and PTGS1, PTGS2, and TNF inhibitor development in RA.

## 1 Introduction

Rheumatoid arthritis (RA) is a chronic inflammatory disease characterized by persistent synovitis, which results in progressive joint damage and even disability [1,2]. RA is regarded as a Bi syndrome in traditional Chinese medicine (TCM), which is characterized by the obstruction of qi and blood in the meridians due to the invasion of external pathogenic wind or cold [3], and Chinese herbal medicines (CHMs) have a long tradition in treating RA [4,5]. *Tripterygium wilfordii* Hook F (TwHF) is the most commonly used TCM CHM for reducing inflammation and alleviating joint pain and swelling. A series of clinical trials revealed that TwHF’s clinical efficacy was comparable to or not inferior to that of available conventional synthetic disease-modifying antirheumatic drugs (csDMARDs). A randomized controlled clinical trial (RCT) reported that TwHF was superior to methotrexate (MTX) monotherapy in active RA, and when combined with MTX, TwHF showed a better curative effect [6], which was subsequently supported by a meta-analysis of RCTs [7], and TwHF extracts have been approved to treat RA in China.

**Figure 1:**
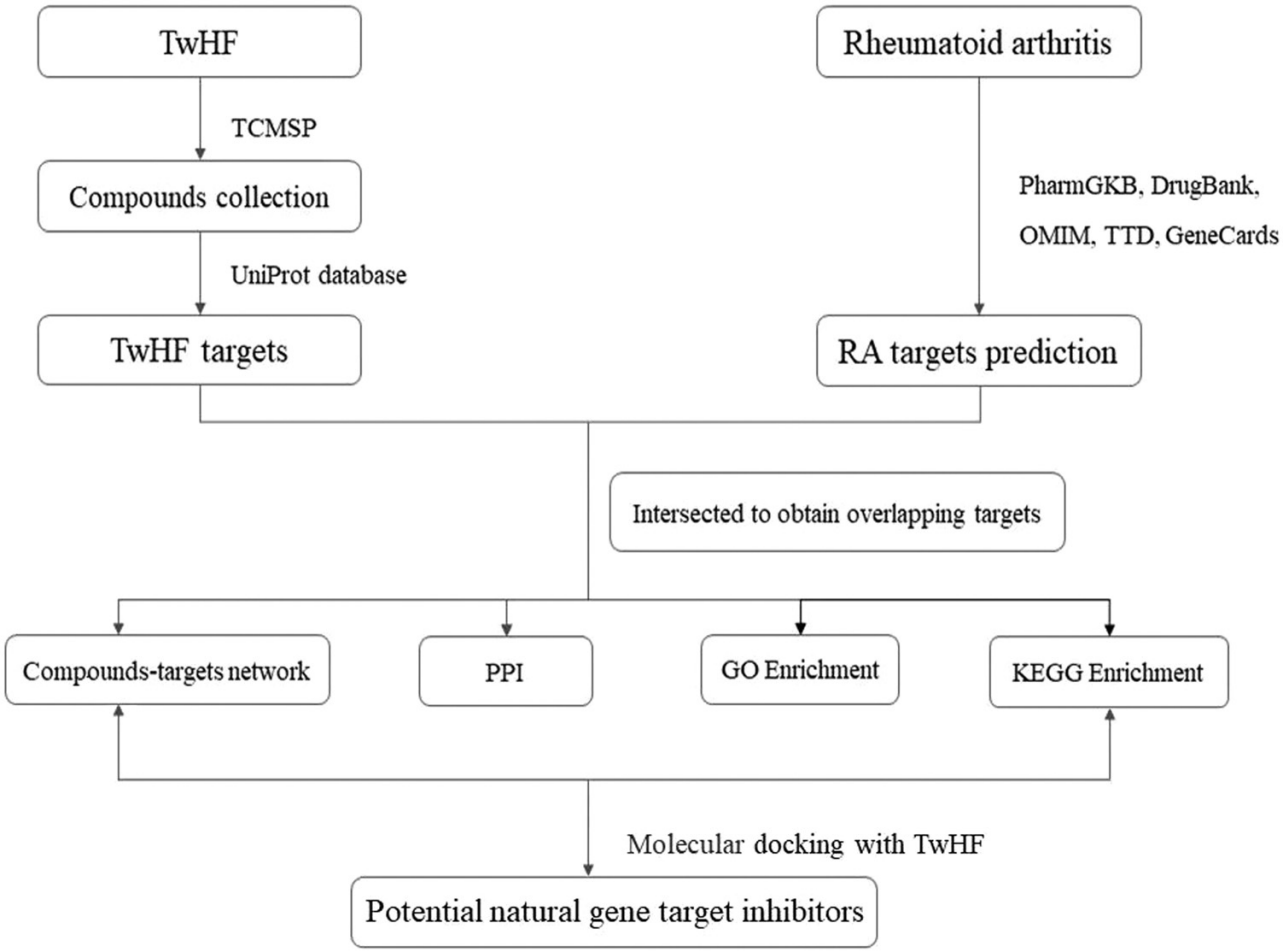
The workflow of gene target inhibitors prediction in RA.

Although animal studies and clinical trials have confirmed the non-steroidal antiinflammatory and immunosuppressive activities of TwHF [8] and inferred that TwHF exerts its effects through promoting T-cell apoptosis, inhibiting proliferation and differentiation of dendritic cell and B cell, reducing the release of pro-inflammatory factors including IL-1β, IL-6, IL-17, and TNF-α [9,10], the precise effective components and the molecular mechanism remain unclear. Moreover, TwHF-related reproductive toxicity, hepatotoxicity, and hematological toxicity are serious concerns that should be closely monitored during treatment [11]. Thus, it is important to deeply understand the pharmacological mechanism of TwHF by exploring the effective components and associated targets. Network pharmacology, which combines network analysis and pharmacology, has been widely applied to study the molecular mechanisms of CHMs and to interpret pharmacological compatibility [12]. In this study, network pharmacology analysis and molecular docking were conducted to explore the anti-RA mechanisms of TwHF (Figure 1).

## 2 Materials and methods

### 2.1 Screening candidate active compounds of TwHF

The absorption, distribution, metabolism, and excretion (ADME) of candidate active compounds of TwHF were screened using the Traditional Chinese Medicine Systems Pharmacology Database and Analysis Platform (TCMSP, available at: https://tcmspw.com/tcmsp.php) [13,14]. According to the previous report [15], ADME screening criteria included oral bioavailability (OB) ≥ 30% and drug-likeness (DL) ≥ 0.18, and compounds without ADME information were excluded. Drug targets of active compounds were obtained from the Universal Protein (UniProt) databases (http://www.uniprot.org) filtered by human species.

### 2.2 RA-related targets screening

The known RA-related targets were obtained through setting “RA” as queries from PharmGKB (https://www.pharmgkb.org), DrugBank (https://go.drugbank.com/), Online Mendelian Inheritance in Man (OMIM, http://www.omim.org), Therapeutic Target Database (TTD, http://db.idrblab.net/ttd), and the Human Gene Databases (https://www.genecards.org/, GeneCards, version 5.7.0, with a relevance score ≥ 1). All databases were filtered for “Homo sapiens” and updated to January 15, 2022.

### 2.3 The intersection of targets of TwHF and RA

The attained targets of active compounds were intersected with RA-related targets to illustrate the overlapping components, which were represented graphically as a Venn diagram drawn by a Venn diagram plotter (https://omics.pnl.gov/software/venn-diagram-plotter).

### 2.4 Topological network construction

Compound–target (C–T) mechanism network was constructed by Cytoscape (https://cytoscape.org/, version 3.8.0) online. In this network, the nodes represent the compounds of TwHF and related targets, while the edges represent the interactions between them.

### 2.5 Protein–protein interaction (PPI) network construction

The overlapping targets of TwHF ingredients and RA obtained above were submitted to the online STRING database (https://string-db.org) to perform PPI analysis, with the minimum required interaction score set as “medium confidence (0.40)” and the disconnected protein nodes excluded. Subsequently, the PPI network was imported into Cytoscape software. Local average connectivity (LAC), closeness centrality (cc), eigenvector centrality (EC), betweenness centrality (BC), degree centrality (DC), and network centrality (NC) were calculated, respectively, by CytoNCA (a plugin in Cytoscape) to seek for the core targets of the PPI network.

### 2.6 Gene ontology (GO) and Kyoto Encyclopedia of Genes and Genomes (KEGG) enrichment analysis

GO enrichment analysis and KEGG pathway enrichment analysis were performed using the statistical software R (version 4.0.5), of which software packages include ggplot2, colorspace, string, DOSE, enrichplot, clusterProfiler, org.Hs.eg.db, and pathview. *P*-values <0.05 with corresponding *Q*-values <0.05 were regarded as significant.

### 2.7 Binding capacity between active ingredients and key target genes by molecular docking

Docking of active ingredients selected from the TwHF-RA-potential target gene network to the key target gene was explored using AutoDock Vina. PDF files for PTGS1 (PDB ID is 1prh), PTGS2 (PDB ID is 5f19), HSP90ABI (PDB ID is 1uym), and TNF (PDB ID is 1tnf) and the active ingredients (files prepared from “TwHF Active Ingredient Database Establishment” section) were uploaded to the AutoDock Vina website. After determining the docking pocket coordinates, molecular docking and conformational scoring were performed using AutoDock. The lower the vina scores are, the most stable is the ligand binding to the receptor, which was used for the preliminary evaluation of the binding activity of the compound to the targets.

## 3 Results

### 3.1 Targets of candidate active compounds of TwHF

Among 144 compounds of TwHF, 51 (Table 1) were selected through ADME criteria (OB ≥ 30 and DL ≥ 0.18). A list of 133 targets was obtained from UniProt databases with filtering for human species and after removing duplicated targets.

**Table 1:** The list of bioactive compounds of TwHF

Mol ID	Compounds	OB (%)	DL
MOL000296	hederagenin	36.91	0.75
MOL003182	(+)-Medioresinol di-*O*-beta-d-glucopyranoside_qt	60.69	0.62
MOL003184	81827-74-9	45.42	0.53
MOL003185	(1*R*,4*aR*,10*aS*)-5-hydroxy-1-(hydroxymethyl)-7-isopropyl-8-methoxy-1,4*a*-dimethyl-4,9,10,10*a*-tetrahydro-3*H*-phenanthren-2-one	48.84	0.38
MOL003187	Triptolide	51.29	0.68
MOL003188	Tripchlorolide	78.72	0.72
MOL003189	WILFORLIDE A	35.66	0.72
MOL003192	Triptonide	67.66	0.70
MOL003196	Tryptophenolide	48.50	0.44
MOL003198	5 alpha-Benzoyl-4 alpha-hydroxy-1 beta,8 alpha-dinicotinoyl-dihydro-agarofuran	35.26	0.72
MOL003199	5,8-Dihydroxy-7-(4-hydroxy-5-methyl-coumarin-3)-coumarin	61.85	0.54
MOL003206	Canin	77.41	0.33
MOL003208	Celafurine	72.94	0.44
MOL003209	Celallocinnine	83.47	0.59
MOL003210	Celapanine	30.18	0.82
MOL003211	Celaxanthin	47.37	0.58
MOL003217	Isoxanthohumol	56.81	0.39
MOL003222	Salazinic acid	36.34	0.76
MOL003224	Tripdiotolnide	56.40	0.67
MOL003225	Hypodiolide A	76.13	0.49
MOL003229	Triptinin B	34.73	0.32
MOL003231	Triptoditerpenic acid B	40.02	0.70
MOL003232	Triptofordin B1	39.55	0.84
MOL003233	Triptofordin B2	107.71	0.76
MOL003234	Triptofordin C2	30.16	0.76
MOL003235	Triptofordin D1	32.00	0.75
MOL003236	Triptofordin D2	30.38	0.69
MOL003238	Triptofordin F1	33.91	0.60
MOL003239	Triptofordin F2	33.62	0.67
MOL003241	Triptofordin F4	31.37	0.67
MOL003242	Triptofordinine A2	30.78	0.47
MOL003244	Triptonide	68.45	0.68
MOL003245	Triptonoditerpenic acid	42.56	0.39
MOL003248	Triptonoterpene	48.57	0.28
MOL003266	21-Hydroxy-30-norhopan-22-one	34.11	0.77
MOL003267	Wilformine	46.32	0.20
MOL003278	Salaspermic acid	32.19	0.63
MOL003279	99694-86-7	75.23	0.66
MOL003280	TRIPTONOLIDE	49.51	0.49
MOL000358	Beta-sitosterol	36.91	0.75
MOL000211	Mairin	55.38	0.78
MOL000422	Kaempferol	41.88	0.24
MOL000449	Stigmasterol	43.83	0.76
MOL002058	40957-99-1	57.20	0.62
MOL003283	(2*R*,3*R*,4*S*)-4-(4-hydroxy-3-methoxy-phenyl)-7-methoxy-2,3-dimethylol-tetralin-6-ol	66.51	0.39
MOL004443	Zhebeiresinol	58.72	0.19
MOL005828	Nobiletin	61.67	0.52
MOL007415	[(2*S*)-2-[[(2*S*)-2-(Benzoylamino)-3-phenylpropanoyl]amino]-3-phenylpropyl] acetate	58.02	0.52
MOL007535	(5*S*,8*S*,9*S*,10*R*,13*R*,14*S*,17*R*)-17-[(1*R*,4*R*)-4-ethyl-1,5-dimethylhexyl]-10,13-dimethyl-2,4,5,7,8,9,11,12,14,15,16,17-dodecahydro-1*H*-cyclopenta[*a*]phenanthrene-3,6-dione	33.12	0.79
MOL009386	3,3′-Bis-(3,4-dihydro-4-hydroxy-6-methoxy)-2*H*-1-benzopyran	52.11	0.54
MOL011169	Peroxyergosterol	44.39	0.82

### 3.2 RA-related targets screening and intersection analysis

**Figure 2:**
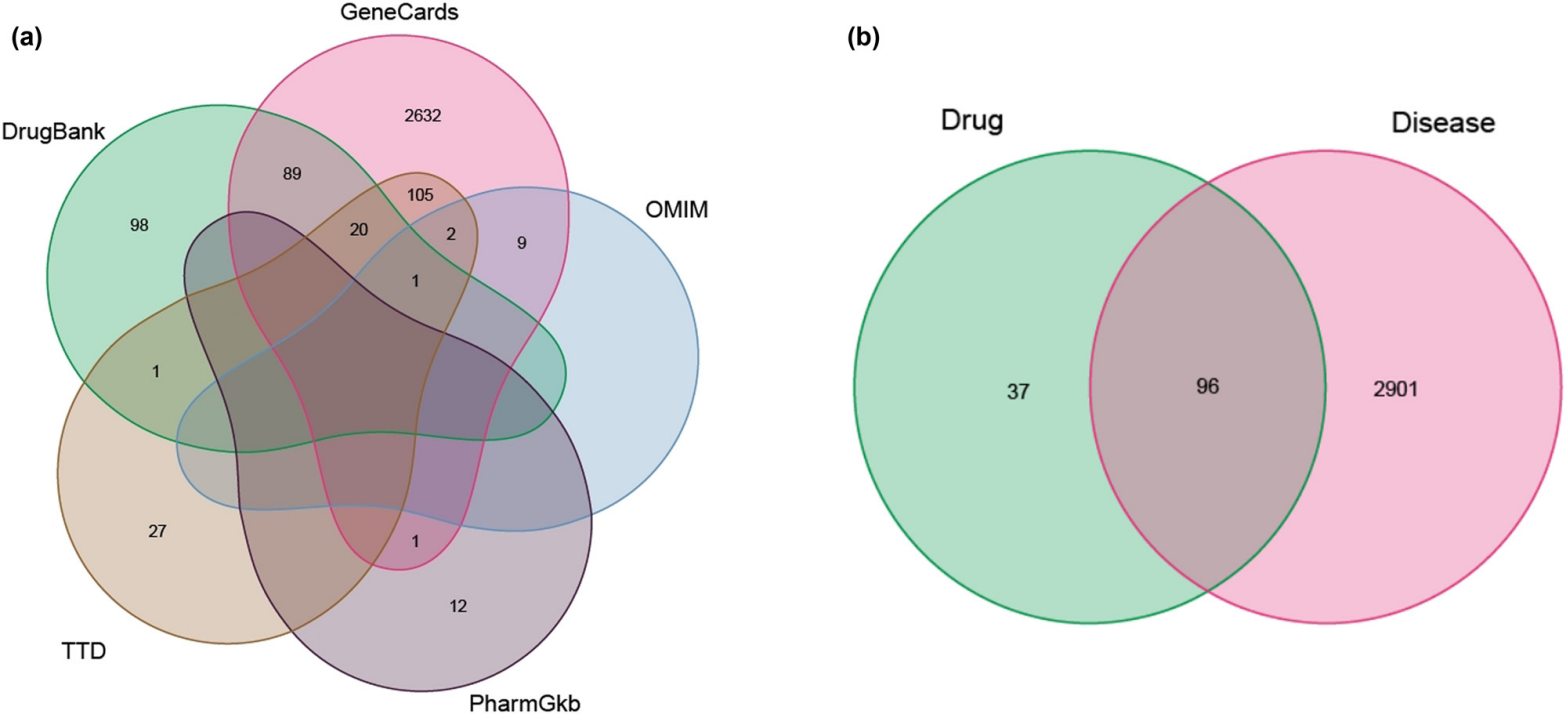
Venn diagram of target screening. (a) A total of 2997 RA-related targets were identified. (b) The intersection of TwHF and RA-related targets. The green circle represents the related targets of TwHF, and the pink circle represents the related targets of RA.

A total of 3,653 RA-related targets were identified from 5 databases, including 13 in PharmGKB, 590 in DrugBank, 27 in OMIM, 164 in TTD, and 2,859 in GeneCards (relevance score ≥ 1). Upon removal of 656 duplicates, 2,997 targets remained (Figure 2a). After being intersected with targets of compounds of TwHF, a total of 96 overlapping targets were illustrated by the Venn diagram (Figure 2b).

### 3.3 Topological network construction

**Figure 3:**
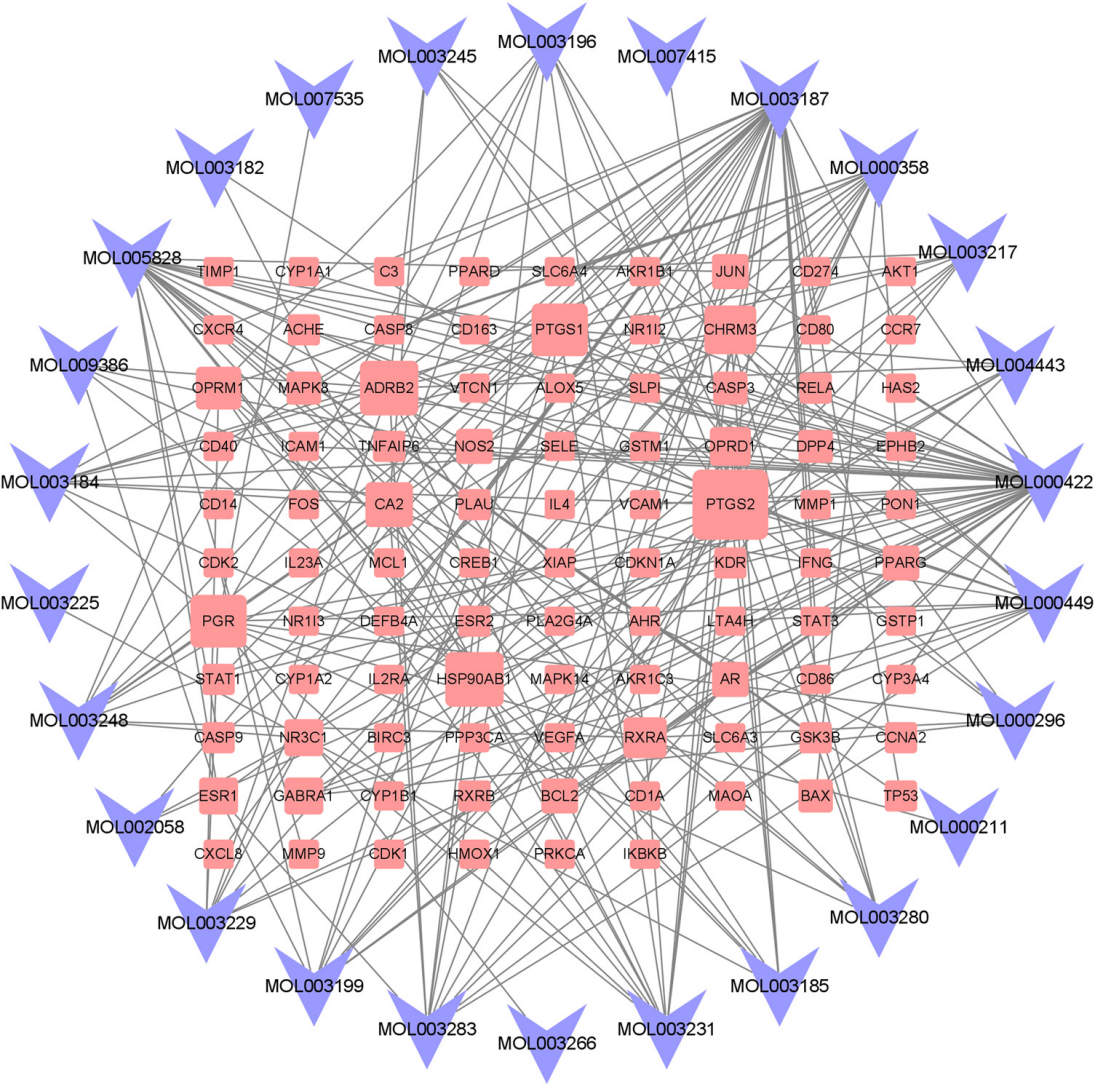
C–T network. Blue V-shape nodes represent active compounds of TwHF and red square nodes represent corresponding targets. Square node size corresponds to the number of edges (degree); the larger the size of the node, the higher its degree (number of interactions).

C–T network of TwHF involved 26 compounds of TwHF and 96 TwHF-RA overlapping target genes, which were represented by 122 nodes and 250 edges. In the C–T network (Figure 3), 9 compounds were associated with greater than or equal to 10 targets (Table 2), including MOL000422 (kaempferol, 40 targets), MOL03187 (triptolide, 33 targets), MOL005828 (nobiletin, 22 targets), MOL000358 (beta-sitosterol, 17 targets), MOL003231 (Triptoditerpenic acid B, 12 targets), MOL000449 (Stigmasterol, 12 targets), MOL003283 [(2*R*,3*R*,4*S*)-4-(4-hydroxy-3-methoxy-phenyl)-7-methoxy-2,3-dimethylol-tetralin-6-ol,12 targets], MOL003199 [5,8-dihydroxy-7-(4-hydroxy-5-methyl-coumarin-3)-coumarin, 10 targets], and MOL003248 (Triptonoterpene, 10 targets).

**Table 2:** TwHF compounds and corresponding targets in the C–T network

Mol ID	Compounds	Targets
MOL000211	Mairin	PGR
MOL000296	Hederagenin	PGR, CHRM3, GABRA1, PTGS1, PTGS2, RXRA
MOL000358	Beta-sitosterol	PGR, PTGS1, PTGS2, HSP90AB1, CHRM3, ADRB2, SLC6A4, OPRM1, GABRA1, BCL2, BAX, CASP9, JUN, CASP3, CASP8, PRKCA, PON1
MOL000422	Kaempferol	NOS2, PTGS1, AR, PPARG, PTGS2, HSP90AB1, DPP4, PGR, ACHE, GABRA1, RELA, IKBKB, AKT1, BCL2, BAX, TNFAIP6, JUN, CASP3, MAPK8, MMP1, STAT1, CDK1, HMOX1, CYP3A4, CYP1A2, CYP1A1, ICAM1, SELE, VCAM1, NR1I2, CYP1B1, ALOX5, HAS2, GSTP1, AHR, NR1I3, PPP3CA, GSTM1, AKR1C3, SLPI
MOL000449	Stigmasterol	PGR, RXRA, PTGS1, PTGS2, SLC6A3, ADRB2, AKR1B1, PLAU, LTA4H, MAOA, CHRM3, GABRA1
MOL002058	40957-99-1	PTGS1, PTGS2, HSP90AB1
MOL003182	(+)-Medioresinol di-*O*-beta-d-glucopyranoside_qt	PTGS2, HSP90AB1
MOL003184	81827-74-9	PTGS1, PTGS2, CHRM3, CA2, OPRD1, PGR, ADRB2, OPRM1, HSP90AB1
MOL003185	(1*R*,4*aR*,10*aS*)-5-hydroxy-1-(hydroxymethyl)-7-isopropyl-8-methoxy-1,4*a*-dimethyl-4,9,10,10*a*-tetrahydro-3H-phenanthren-2-one	CHRM3, PTGS2, CA2, OPRD1, OPRM1, NR3C1, ADRB2, HSP90AB1
MOL003187	Triptolide	RELA, STAT3, VEGFA, BCL2, FOS, CDKN1A, PLAU, TNFAIP6, JUN, CASP3, TP53, MAPK8, PTGS2, STAT1, CXCL8, MCL1, IL2RA, IFNG, IL4, XIAP, DEFB4A, CD80, CD86, CXCR4, BIRC3, CD274, IL23A, CCR7, CD1A, CD40, CD14, C3, VTCN1
MOL003196	Tryptophenolide	CHRM3, PTGS2, CA2, RXRA, OPRD1, PGR, ADRB2, OPRM1, HSP90AB1
MOL003199	5,8-Dihydroxy-7-(4-hydroxy-5-methyl-coumarin-3)-coumarin	NOS2, PTGS1, ESR1, AR, PPARG, PTGS2, KDR, DPP4, HSP90AB1, CDK2
MOL003217	Isoxanthohumol	NOS2, ESR1, PTGS2, KDR, ADRB2, HSP90AB1, PTGS1, PPARD
MOL003225	Hypodiolide A	NR3C1
MOL003229	Triptinin B	CHRM3, PTGS2, CA2, RXRA, PGR, ADRB2, OPRM1, NR3C1, RXRB
MOL003231	Triptoditerpenic acid B	PTGS1, CHRM3, PTGS2, CA2, RXRA, OPRD1, PGR, ADRB2, OPRM1, NR3C1, HSP90AB1, RXRB
MOL003245	Triptonoditerpenic acid	CHRM3, PTGS2, CA2, OPRD1, ADRB2
MOL003248	Triptonoterpene	PTGS1, CHRM3, PTGS2, CA2, RXRA, ACHE, PGR, ADRB2, OPRM1, NR3C1
MOL003266	21-Hydroxy-30-norhopan-22-one	PGR
MOL003280	TRIPTONOLIDE	CHRM3, PTGS2, CA2, OPRD1, PGR, ADRB2, OPRM1
MOL003283	(2*R*,3*R*,4*S*)-4-(4-Hydroxy-3-methoxy-phenyl)-7-methoxy-2,3-dimethylol-tetralin-6-ol	ESR1, AR, PPARG, PTGS2, CA2, ADRB2, ESR2, MAPK14, GSK3B, HSP90AB1, CCNA2, PTGS1
MOL004443	Zhebeiresinol	PTGS1, PTGS2, RXRA, ADRB2, GABRA1, HSP90AB1
MOL005828	Nobiletin	NOS2, PTGS1, ESR1, AR, PPARG, PTGS2, ESR2, DPP4, HSP90AB1, GSK3B, BCL2, BAX, CASP9, MMP9, JUN, TP53, MAPK8, TIMP1, CREB1, PLA2G4A, CD163, EPHB2
MOL007415	[(2*S*)-2-[[(2*S*)-2-(benzoylamino)-3-phenylpropanoyl]amino]-3-phenylpropyl] acetate	PTGS2
MOL007535	(5*S*,8*S*,9*S*,10*R*,13*R*,14*S*,17*R*)-17-[(1*R*,4*R*)-4-ethyl-1,5-dimethylhexyl]-10,13-dimethyl-2,4,5,7,8,9,11,12,14,15,16,17-dodecahydro-1*H*-cyclopenta[*a*]phenanthrene-3,6-dione	PGR
MOL009386	3,3′-bis-(3,4-Dihydro-4-hydroxy-6-methoxy)-2*H*-1-benzopyran	ESR1, PTGS2, ADRB2, HSP90AB1, CCNA2

### 3.4 PPI network construction

**Figure 4:**
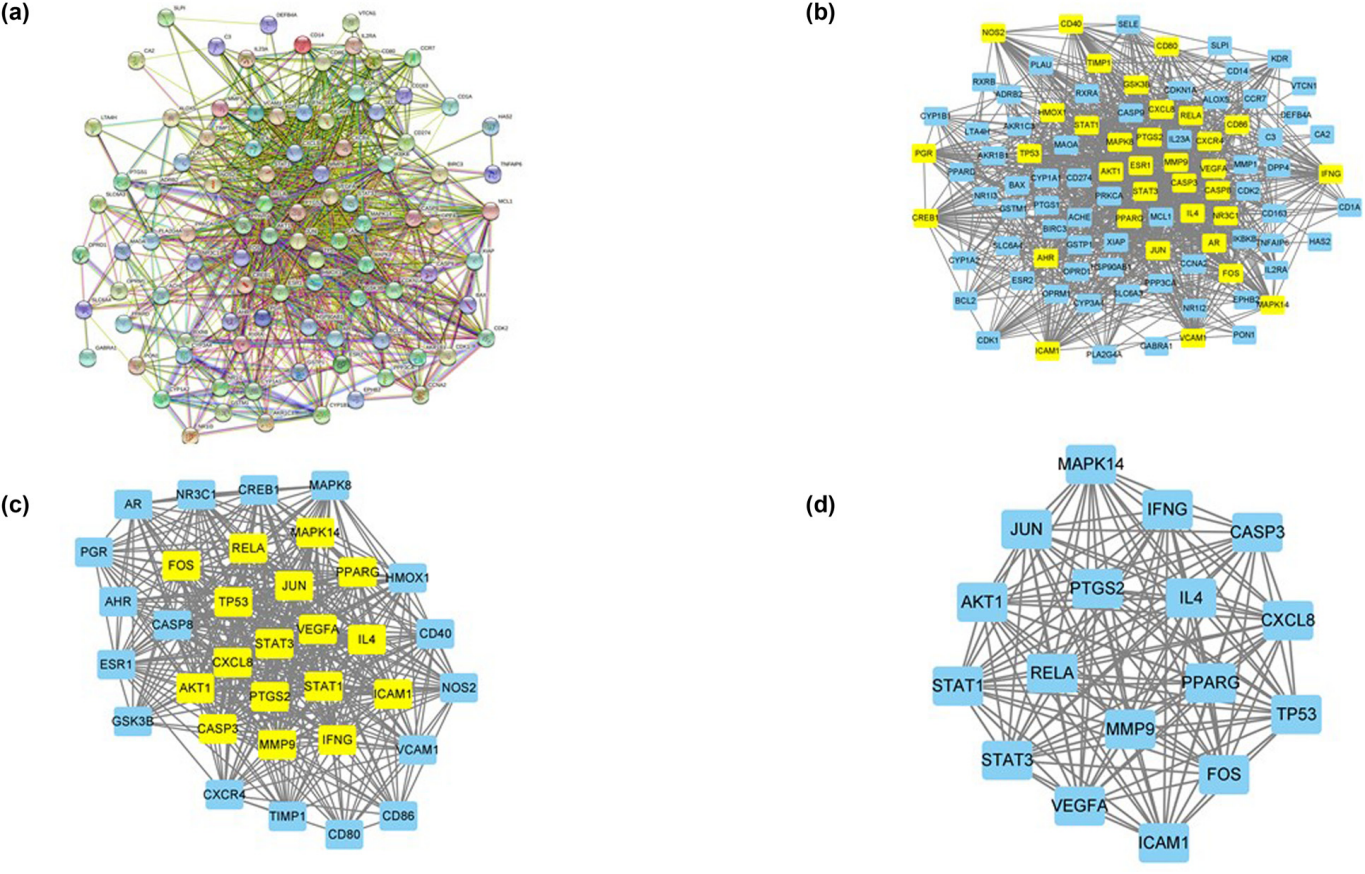
The PPI network of TwHF-RA. (a) The PPI network of TwHF-RA targets was obtained from STRING. (b) The PPI network after being subjected to Cytoscape. Yellow squares represent significant targets. (c) The PPI network of significant targets extracted from B. Yellow squares represent significant targets. (d) The PPI network of crucial TwHF targets for RA treatment extracted from (c).

The PPI network contained 95 nodes and 1161 edges with a hiding disconnected node (Figure 4a). After being imported into Cytoscape (Figure 4b), calculated with CytoNCA, and filtered by LAC > 15.55555556, cc > 0.537142857, EC > 0.079239734, BC > 28.75112417, DC > 21, and NC > 16.50735294, a new PPI network (Figure 4c, 34 nodes and 438 edges) was constructed. Subsequently, the new PPI network was re-calculated with CytoNCA and re-filtered with LAC > 21.40923077, cc > 0.8149390245, EC > 0.169334501, BC > 5.518303103, DC > 25.5, and NC > 23.78119305, and it was further refined to another PPI network (Figure 4d, 17 nodes and 135 edges) with 18 targets potentially playing a crucial role retained, including CXCL8 (*n* = 16), CXCL6 (*n* = 16), STAT3 (*n* = 16), STAT1 (*n* = 16), JUN (*n* = 16), PPARG (*n* = 16), TP53 (*n* = 16), IL14 (*n* = 16), MMP9 (*n* = 16), VEGFA (*n* = 16), RELA (*n* = 16), CASP3 (*n* = 16), PTGS2 (*n* = 16), IFNG (*n* = 16), AKT1 (*n* = 16), FOS (*n* = 16), ICAM1 (*n* = 15), and MAPK14 (*n* = 15).

### 3.5 GO and KEGG enrichment analyses

**Figure 5:**
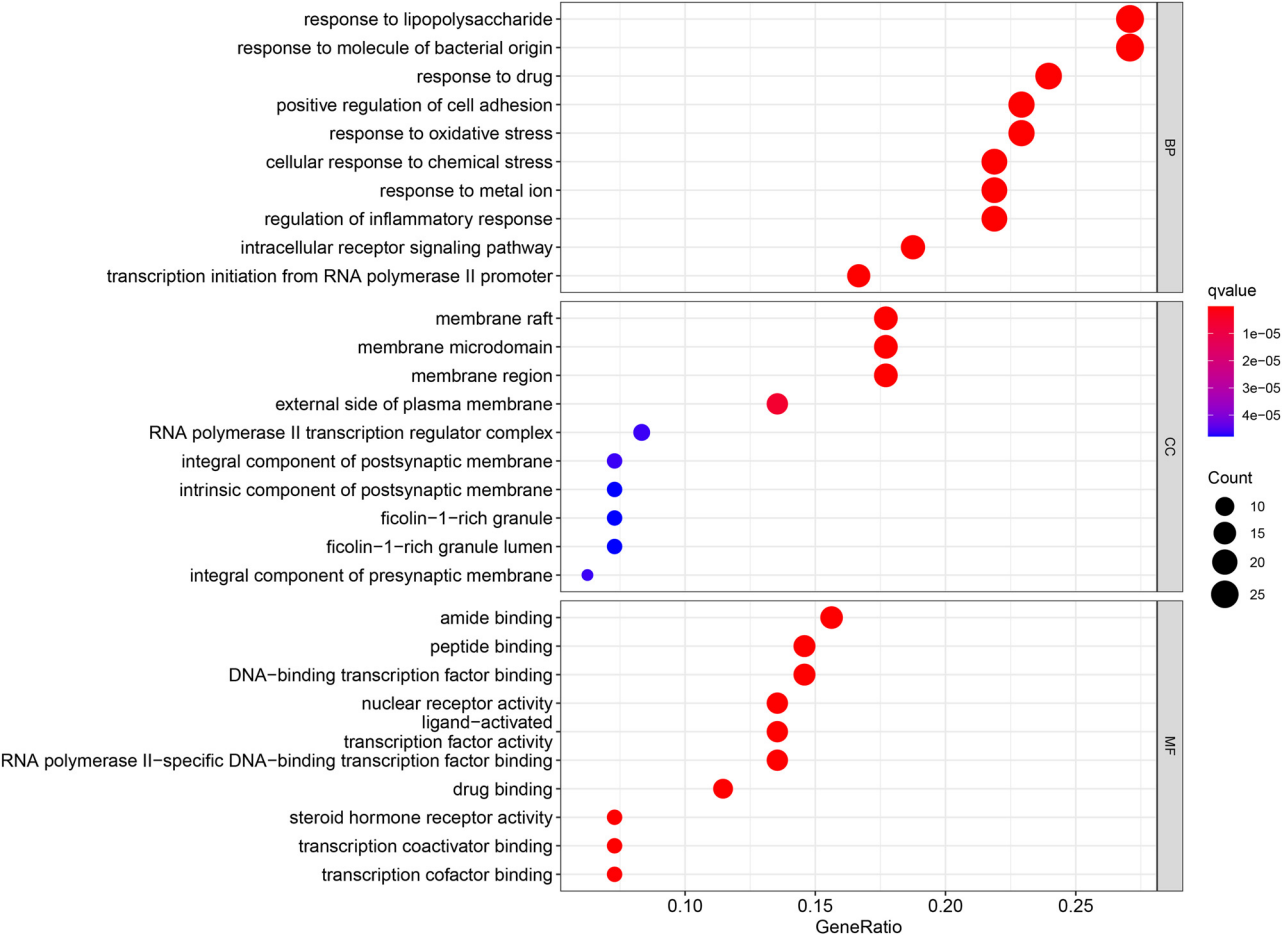
GO enrichment analysis of potential targets. The top 10 terms (*q* < 0.05) of each part (BP, CC, and MF) are shown. The vertical axes represent the enriched GO pathways, while the horizontal axes represent the GeneRatio of each GO pathway. Bubble size reflects the number of genes involved and bubble color reflects the *q* value; the redder the bubble, the smaller the *q* value.

**Figure 6:**
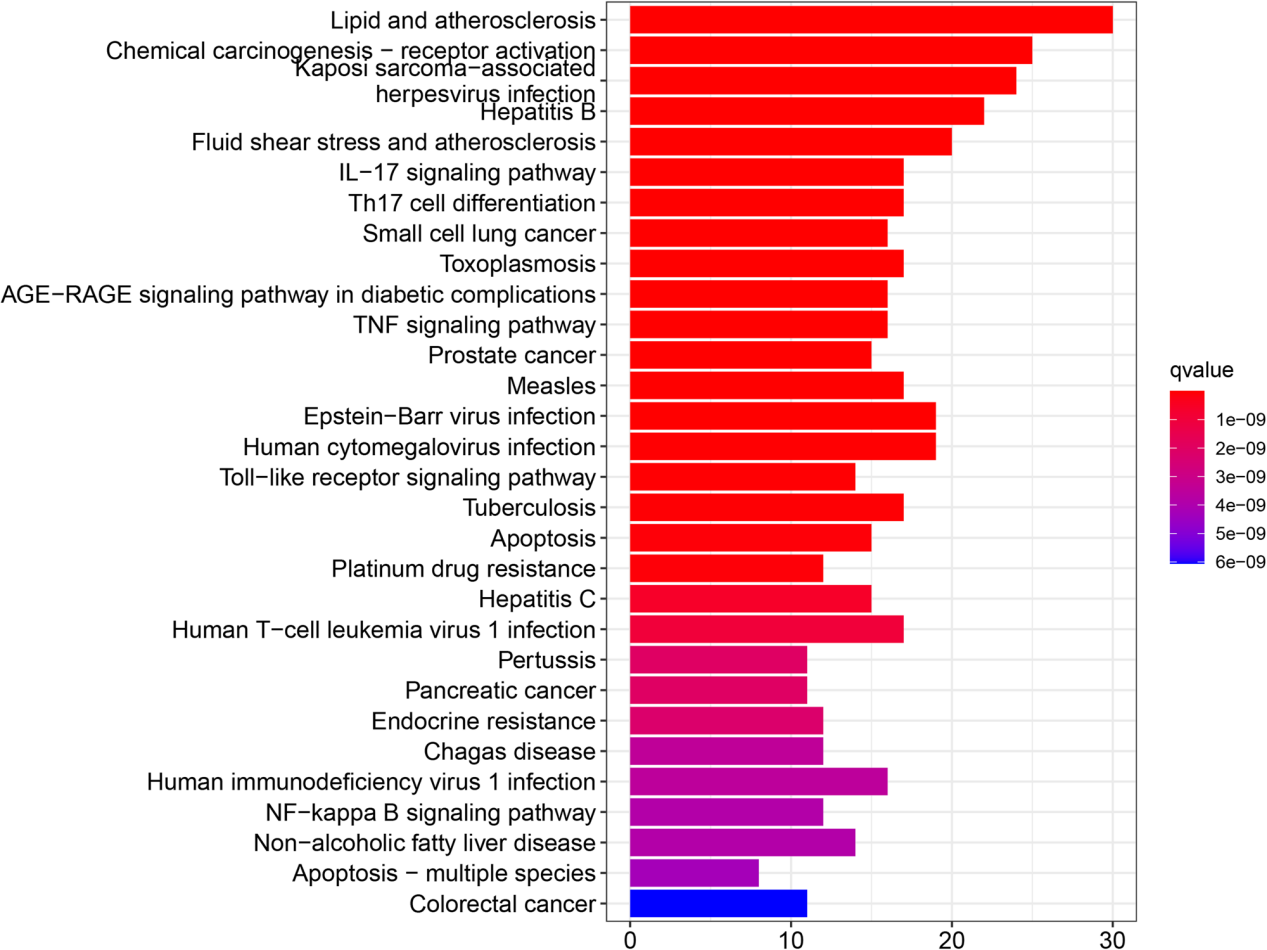
Scatterplot of enriched KEGG pathways. The vertical ax represents the enriched KEGG pathways. The bar length represents the number of enriched targets in a certain KEGG pathway; the longer the bar, the more the number of enriched targets. Only the top 30 terms are listed here. And the bar color reflects the *q* value; the redder the color, the smaller the *q* value.

In Figure 5, GO enrichment analysis showed the top 10 significantly enriched GO terms in each part. According to the biological processes (BP), potential gene targets were mainly concentrated in response to lipopolysaccharide, response to molecules of bacterial origin, response to drugs, and so on. Based on cell components (CC), the targets were mainly enriched in membrane raft, membrane microdomain, membrane region, and so on. In terms of molecular function (MF), potential targets were mainly related to amide binding, peptide binding, DNA-binding transcription factor binding, and so on. KEGG enrichment analysis was performed to identify the signaling pathways of these targets. The top 30 most potential signaling pathways are shown in Figure 6 according to *q* value. KEGG results suggested that TwHF alleviated RA by regulating multiple signaling pathways, which were closely related to lipid and atherosclerosis, chemical carcinogenesis-receptor activation, Kaposi sarcoma-associated, herpesvirus infection, hepatitis B, fluid shear stress and atherosclerosis, IL-17 signaling pathways, Th17 cell differentiation, small cell lung cancer, toxoplasmosis, AGE-RACE signaling pathway in diabetic complications, TNF signaling pathway, and so on, and these pathways are mainly associated with metabolism, cancer, immunity, infection, inflammatory response, and oxidative stress.

### 3.6 Binding capacity between the active ingredient triptolide and PTGS1, PTGS2, HSP90ABI, and TNF by molecular docking

**Figure 7:**
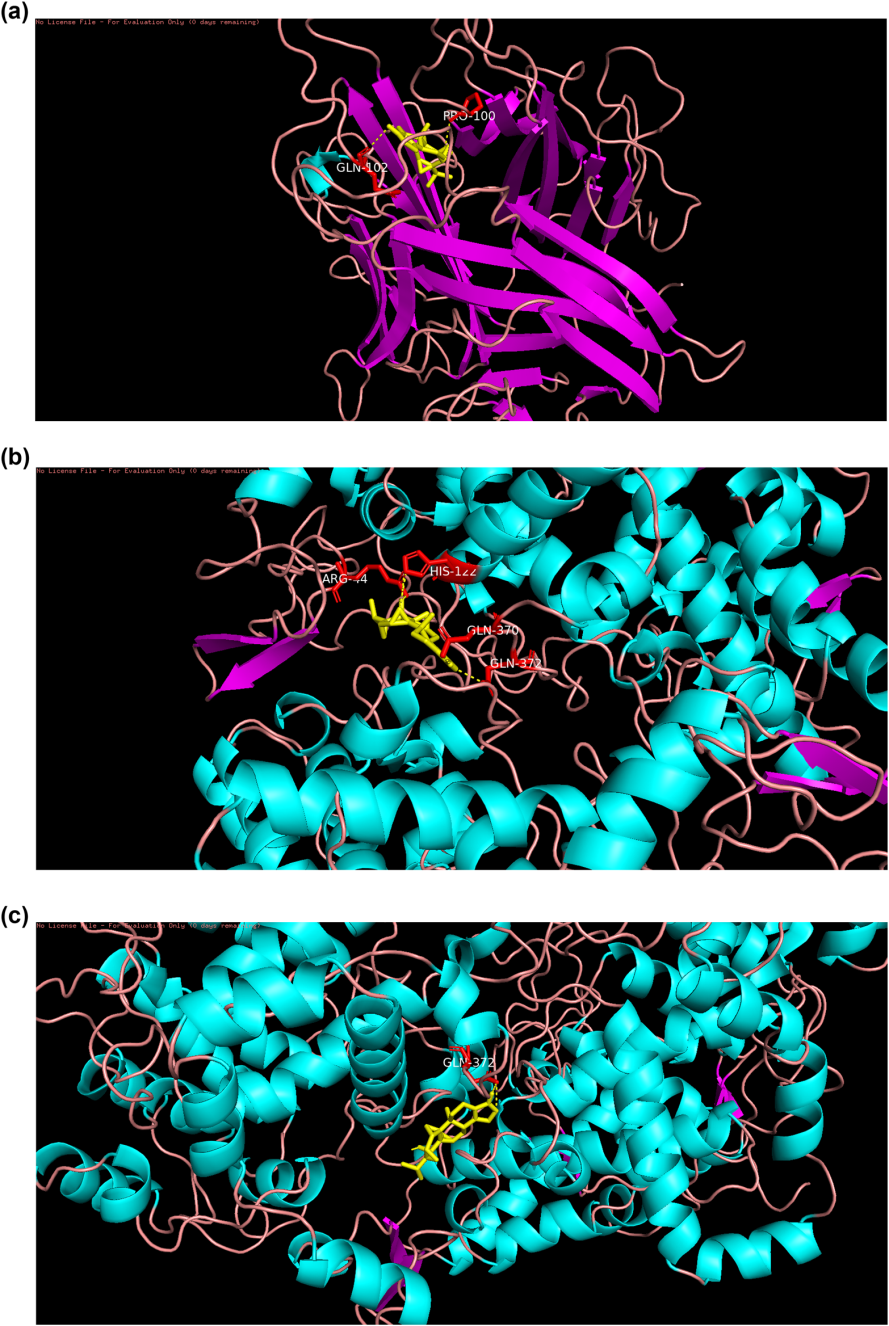
The 3D map of the binding of triptolide. (a) TNF, (b) PTGS2, and (c) PTGS1.

**Figure 8:**
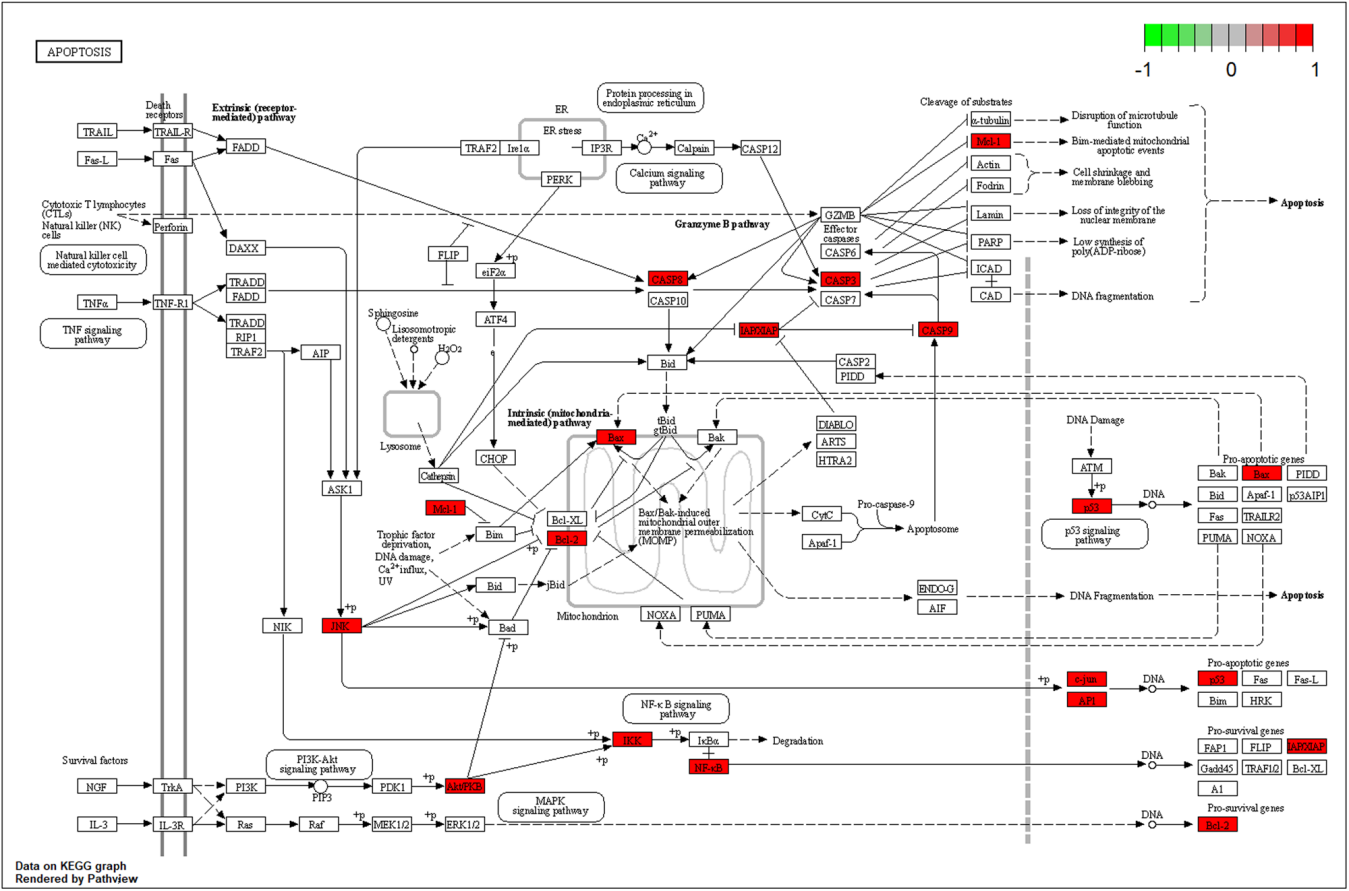
The TNF-α signaling pathway of potential target genes of TwHF in RA. Arrows indicate upstream and downstream relationships between genes. The red is a TwHF target gene in the network.

**Figure 9:**
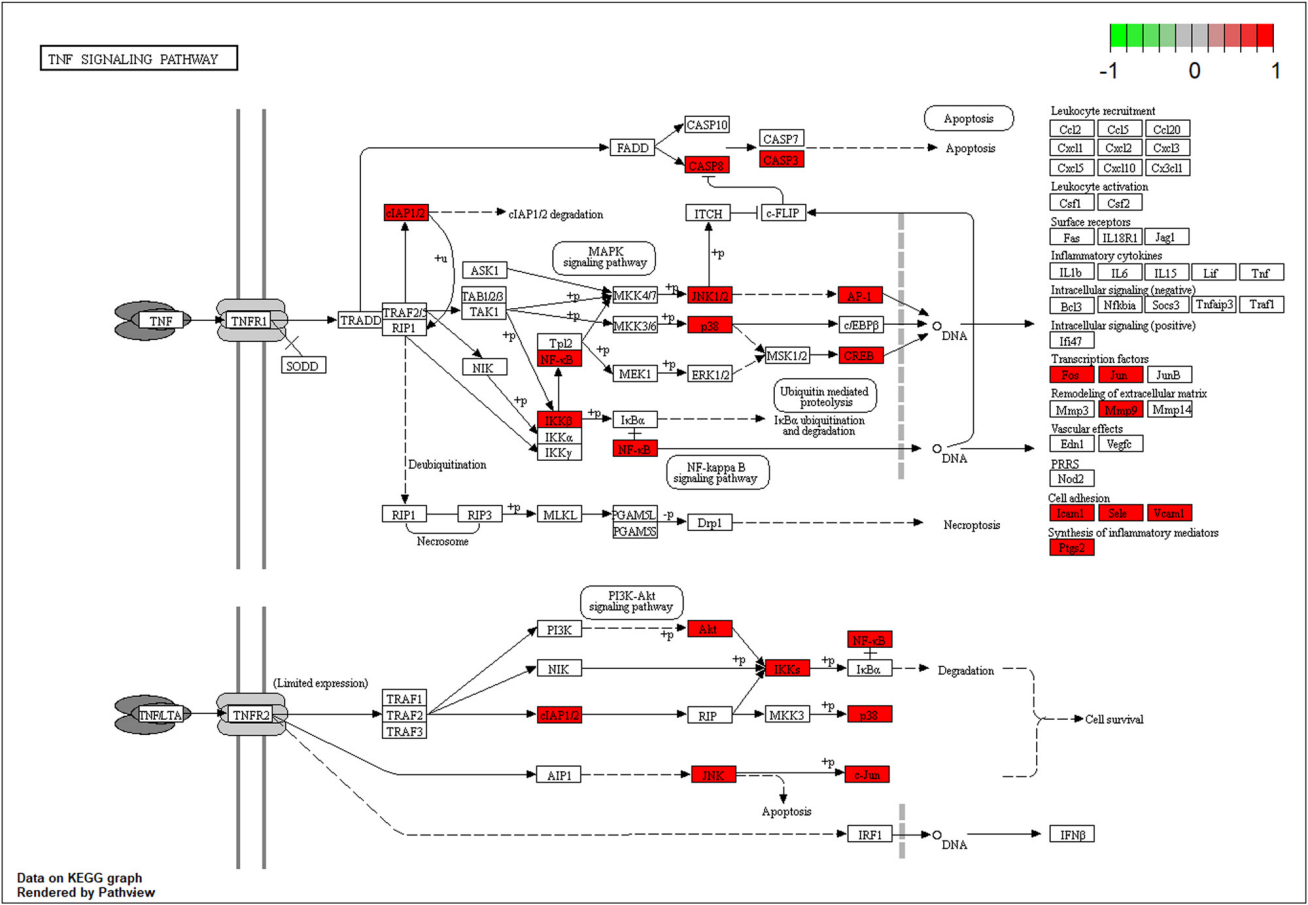
The apoptosis signaling pathway of potential target genes of TwHF in RA. Arrows indicate upstream and downstream relationships between genes. The red represents the TwHF target gene in the network.

Active component triptolides selected from the TwHF-RA-potential target gene network bind PTGS1, PTGS2, HSP90ABI, and TNF to varying degrees (Table 3). Lower vina scores indicate a stronger and stable interaction between the compound and receptor. Triptolide had the strongest and most stable binding affinity toward TNF, PTGS2, and PTGS1. These results suggest that triptolide may be the most appropriate material basis for a natural TNF, PTGS2, and PTGS1 inhibitor. The 3D map of the binding of PTGS1, PTGS2, HSP90ABI, and TNF to triptolide is shown in Figure 7a–c. Additionally, the vital TNF-α signaling pathway is shown in Figure 8. The vital apoptosis signaling pathway is shown in Figure 9.

**Table 3:** Molecular docking parameters and results of Triptolide in TwHF binding with TNF, PTGS2, PTGS1, and TNF

Target gene	Vina scores	Center		
		*x*	*y*	*z*
TNF	−9.5	25.362	68.355	51.594
PTGS2	−9.3	34.493	61.190	59.717
PTGS1	−9.0	34.870	11.640	190.336
HSP90ABI	−8.3	13.754	25.415	28.695

## 4 Discussion

RA affects about 0.5–1% of the population with a higher mortality rate, resulting in a heavy economic, emotional, and social burden for both the individuals and their families [16]. Currently, csDMARDs and biological agents remain the mainstream for clinical treatment in RA [1]. However, the lack of adequate response of csDMARDs and high prices of biological therapy remain difficult problems to be solved in the clinic. TCM has a long history in the treatment of RA, and many studies have shown that TwHF had good clinical effectiveness in RA treatment with considerably low costs [8]. However, in recent years, almost more than 300 kinds of ingredients have been identified with modern technology from TwHF [17], of which the specific dominating anti-rheumatic functional components and the underlying mechanisms still require further exploration. Given this, we conducted this network pharmacology analysis to determine the possible mechanisms of TwHF in RA.

In the Compounds-targets network analysis of TwHF, representative ingredients with a high degree include kaempferol, triptolide, nobiletin, and beta-sitosterol. Kaempferol has multiple pharmacological effects such as antitumor, anti-inflammatory, anti-infection, and antioxidant. And animal studies showed that the oral administration of kaempferol showed distinct anti-arthritis effects in collagen-induced arthritis (CIA) model mice by reshaping the intestinal microbial community and modulating the microbiota-mediated metabolism of tryptophan, fatty acids, and secondary bile acids and energy production [18]. Triptolide is one of the most studied and characterized components of TwHF. Triptolide may possess an anti-RA effect by downregulating the angiogenic activators and inhibiting the activation of mitogen-activated protein kinase downstream signal pathway [19], decreasing the production of TNF-alpha, IL-1beta, and IL-6 [20,21], and preventing the bone destruction and inhibit osteoclast formation by reducing the expression of receptor activator of NF-kappa B ligand (RANKL) and RANK and increasing the expression of osteoprotegerin (OPG) [22]. Nobiletin, belonging to flavonoids, has the potential of anti-RA by inhibiting IL-1-induced prostaglandin E2 (PGE2) production, pannus formation, and matrix degradation of rabbit articular cartilage [23], suppressing aggrecanase-mediated degradation of aggrecan in CIA mice [24], and repressing the angiogenesis and inflammatory infiltration by down-regulating the protein expression level of the p38/NF-kappa B signaling pathway in the synovium of CIA rats [25]. Beta-sitosterol, a kind of phytosterol, has various biological activities. It could augment M2 polarization, reduce the levels of collagen-specific antibodies, and inhibit the production of pro-inflammatory cytokines in CIA mice [26]. It also showed the antiarthritic effect via suppression of NF-kB and activation of Heme Oxygenase-1 (HO-1)/nuclear factor erythroid 2-related factor 2 (Nrf2) pathway [27].

In the PPI network, a total of 18 potential targets with a high degree were inferred as playing a crucial role in the treatment of RA. CXCL8 (IL-8) was reported to increase in RA, and it exerts a role in RA inflammation by triggering neutrophils via its specific GPCRs (G-protein-coupled receptors), CXCR1 (CXC chemokine receptor 1), and CXCR2 [16,28,29]. CXCL6 also contributes to neutrophil recruitment and is associated with pathways involved in inflammation and apoptosis [30]. The JAK/STAT signaling was one of the most important downstream inflammatory pathways in RA, and suppression of phosphorylation of STAT3 and STAT1 contributed to RA control [31–34]. JUN signaling was related to vascular remodeling and enhanced collagenase gene expression in RA [35,36]. PPARG could inhibit the expression of inflammatory factors such as TNF-α and IL-1, and a reduction in PPARG may be related to systemic inflammation and ectopic lipid deposition into skeletal muscle and liver [37,38]. TP53, originally known as a tumor suppressor, has been reported to potentially have pro-tumorigenic effects via increased inflammation or anti-apoptotic mechanisms [39,40]. Elevated levels of MMP9 are a common feature in autoimmune diseases such as RA. MMP9 could influence the inflammatory process positively through the activation of pro-IL-1β [41] and increase arthritis by degrading anti-inflammatory factors, activating inflammatory factors, or promoting the migration of inflammatory cells [42]. Upregulation of VEGFA is involved in tumor growth, metastasis, and angiogenesis, VEGFA gene polymorphism was also reported to be linked with RA risk and activity [43–45]. RelA (NF-κB p65) and MAPK family members belong to inflammatory signaling molecules and are important for pro-inflammatory response and cell survival [46,47]. IFNG dominates in Th1 immunity and contributes to RA inflammation [48,49]. Other key targets, such as IL14 CASP3, PTGS2, AKT1, FOS, and ICAM1, are involved in inflammatory or pro-tumorigenic signaling pathways, playing a crucial role in cytokine production, anti-oxidation, cell growth, proliferation, or differentiation [50–58].

GO and KEGG enrichment analyses indicated that the screened targets were mainly enriched in modulating signaling pathways associated with tumor, infection, and Th17 and Th1 immunities. RA, tumor, and infection often share some overlapping pathways, which are important both in the carcinogenesis and inflammation, such as JAK/STAT, PI3K/Akt, MAPK signaling pathways, and so on [59–65]. Infection, such as Epstein-Barr virus (EBV) infection, could increase the CD25 + B-cell subset and induce immunoglobulin production, contributing to the pathogenesis of RA [66,67]. Th1 immunity is an absolute requirement for the clearance of intracellular infection and tumor cells, and also, it is crucial in the pathogenesis of autoinflammatory diseases such as RA. INFG and TNF were the primary inflammatory cytokines and effectors in Th1 immunity [68,69] and anti-TNF therapies have been the first choice of biological treatment in RA in recent years [70,71]. IL-17, a signature cytokine of Th17 immunity, can enhance the secretion of several other inflammatory factors such as tumor necrosis factor-alpha (TNF-α), and IL-1β that result in angiogenesis and osteoclastogenesis in RA [68,72]. Treatment against IL-17 was reported to exhibit a significant clinical efficacy in patients with active RA [73].

In this study, we explored the anti-RA mechanisms of TwHF with network pharmacological analysis and molecular docking. Kaempferol and triptolide might be the most proper bioactive compounds of TwHF in treating RA. CXCL8, CXCL6, STAT3, STAT1, JUN, PPARG, TP53, IL14, MMP9, VEGFA, RELA, CASP3, PTGS2, IFNG, AKT1, FOS, ICAM1, and MAPK14 constituted the core targets of TwHF treatment. Enrichment analysis elaborated multifunctional synergetic mechanisms of TwHF in treating RA, especially including IL-17 signaling pathways, Th17 cell differentiation, and TNF signaling pathways, which were the key pathogenic factors in RA. Our finding indicated that triptolide has excellent therapeutic effects on RA and inhibits TNF, PTGS2, and PTFS1. However, there are some limitations to this study. First, there exist many variable factors in CHMs, such as the site of drug origin, drug dose, dosage form, and so on. Therefore, the potential confounding bias was inevitable. Moreover, although it was referred that kaempferol and triptolide might be the candidate compounds of TwHF in treating RA through the reduction of inflammatory factors, suppression of chemotaxis of immune cells, and inhibition of angiogenesis, their precise effects and mechanisms still require further validation. Nevertheless, this study provides an innovative approach for exploring multiple mechanisms in TCM.

## 5 Conclusion

In summary, TwHF plays an important role in the treatment of RA with various targets and signaling pathways. The biological functions and signaling pathways of the TwHF active ingredients on RA target genes were investigated by the network pharmacology approach. In particular, triptolide, with optimal molecular binding to TNF, PTGS2, and PTFS1, was obtained by the molecular binding assay and can be researched as the most appropriate TNF, PTGS2, and PTGS1 inhibitors. These findings will further reveal the molecular biological mechanism of TwHF in the treatment of RA and provide a theoretical basis for the clinical treatment of RA.

## Abbreviations

RA: rheumatoid arthritis
TCM: traditional Chinese medicine
CHM: Chinese herbal medicines
TwHF: *Tripterygium wilfordii* Hook F
csDMARD: conventional synthetic disease-modifying antirheumatic drug
